# Advances in digital CBT: where are we now, and where next?

**DOI:** 10.1017/S1754470X22000423

**Published:** 2022-10-19

**Authors:** Graham R. Thew, Alexander Rozental, Heather D. Hadjistavropoulos

**Affiliations:** 1 Department of Experimental Psychology, University of Oxford, Oxford, UK; 2 Oxford Health NHS Foundation Trust, Oxford, UK; 3 Department of Psychology, Uppsala University, Uppsala, Sweden; 4 Department of Clinical Neuroscience (CNS), Karolinska Institutet, Stockholm, Sweden; 5 Great Ormond Street Hospital Institute of Child Health, UCL, London, UK; 6 Department of Psychology, University of Regina, Regina, Canada

**Keywords:** apps, CBT, cCBT, IAPT, internet interventions, remote treatment

## Abstract

Digital CBT refers to the use of digital tools, platforms or devices to deliver or enhance cognitive behavioural therapy assessment, formulation, treatment, training and supervision. The ‘Advances in Digital CBT’ special issue aimed to document examples of innovative digital CBT practice in this rapidly developing field. In this paper, we have briefly summarised and synthesised the advances demonstrated in this group of articles. These include developments in our understanding of mental health apps, the use of digital tools as an adjunct to therapy, the effectiveness of remotely delivered CBT in routine clinical practice, our understanding of user experiences and involvement, and in digital CBT research methods. We consider the extent of current knowledge in these areas and identify where gaps in evidence lie and how the field could be taken forward to address these. Lastly, we reflect on the broader digital CBT picture and offer our suggestions of six key directions for future research: using robust study designs to evaluate and optimise digital tools; translating and culturally adapting digital tools and practices; understanding and addressing digital exclusion; exploring, reporting and addressing possible negative effects; improving user involvement in design and evaluation; and addressing the implementation gap for digital tools. We suggest that further advances in these areas would be of particular benefit to the digital CBT field.

## Introduction

Digital CBT can be defined broadly as the use of digital tools, platforms or devices to deliver or enhance cognitive behavioural therapy assessment, formulation, treatment, training and supervision. Research and clinical practice in this area have been ongoing for more than 30 years, with advances in technology facilitating new and innovative approaches to our CBT activities. More recently, practitioners across the world have experienced a huge shift in the implementation of digital CBT in response to the COVID-19 pandemic. The use of technology to deliver sessions remotely, or to support clients in other ways than the traditional ‘weekly face-to-face session’ model has been vital during this time, and the flexibility and reach that these methods provide suggests they will continue to be a large part of treatment delivery in future.

The aim of the ‘Advances in Digital CBT’ special issue was to document examples of innovative digital CBT practice and evaluate how such advances might contribute to this field, and to CBT in general. In this paper we briefly synthesise the findings of these studies and reflect on the broader advances in digital CBT that they suggest. We will also consider the gaps and limitations identified in this current work and how they could be addressed. Extending this further, we offer our thoughts on the broader areas of digital CBT where advances are needed, highlighting six key directions for future research that would offer most promise for further developing the field.

## Articles overview

We will begin with a brief overview of the articles in the special issue and the topics they address. Three articles focus on mental health-related apps. These include an account of the co-design procedures used in the development of a CBT app for adolescents with depression and anxiety (Li *et al*., 2022), a review of apps that aim to complement clinical treatment by supporting assessment/diagnosis, treatment planning, treatment fidelity monitoring, or outcome tracking (Pacheco and Scheeringa, 2022), and a review of apps implementing cognitive restructuring techniques (Erhardt *et al*., 2022). Three articles focus on remote treatment delivery, including an evaluation of the effectiveness of remote therapy in two Improving Access to Psychological Therapies (IAPT) services in London (Nguyen *et al*., 2022), an evaluation of the outcomes of CBT delivered via videoconferencing for young people treated in routine clinical practice (Porter *et al*., 2022), and a qualitative investigation of patient and therapist experiences of videoconferencing (Song and Foster, 2022). Two articles examine digital adjuncts to treatment, one providing an evaluation of user experiences of a CBT-based online peer support platform (Browne *et al*., 2022), and the other examining the uptake and perceptions of a self-guided booster lesson among university students who had completed a course of internet-delivered CBT (Patterson *et al*., 2022).

## What advances have been made, and how can these be extended?

We now consider the advances that these articles suggest and what these tell us about the progression of digital CBT. We also reflect on potential gaps and limitations and consider how these could be addressed to extend these advances further.

### Apps and adjuncts to treatment

One clear advance reflected in this group of articles is in the development of apps, specifically in what some apps can now do in terms of delivering CBT techniques (Erhardt *et al*., 2022; Li *et al*., 2022), or in their use as an adjunct to therapist-led treatment formats (Pacheco and Scheeringa, 2022). This represents a valuable route towards making CBT more accessible and widely available, particularly for people or groups who may be less likely to access traditional mental health services. However, although high-quality mental health apps exist, with engaging content and strong fidelity to evidence-based treatment principles, the app reviews (Erhardt *et al*., 2022; Pacheco and Scheeringa, 2022) demonstrate how these may be hard to find given the sheer volume of mental health apps available. Pacheco and Scheeringa (2022) found 722 apps in their initial search for mental health apps, of which only 163 met their criteria for having a substantive clinical element. Erhardt *et al*. (2022) aimed to identify apps implementing cognitive restructuring, but even this more targeted search returned 256 apps, of which only 15 implemented the cognitive restructuring technique. These studies also note that the information provided about apps is often insufficient to fully understand what the app provides and how. Independent platforms that rate and evaluate mental health apps (such as One Mind PsyberGuide, and the mHealth Index and Navigation Database ‘MIND’) are available, though may not be exhaustive.

Reviews such as Pacheco and Scheeringa (2022) and Erhardt *et al*. (2022) are valuable in that they provide thorough and fine-grained analyses of what specific apps provide. The study of Erhardt *et al*. (2022) highlights that cognitive restructuring apps include most core elements of the technique, although they vary regarding additional elements such as asking for emotion intensity ratings. They make the interesting point that different apps may be more helpful depending on the specific needs of the user and note that improvements are needed due to variability in privacy protections and features to support user engagement.

Studies such as Browne *et al*. (2022), Pacheco and Scheeringa (2022) and Patterson *et al*. (2022) reflect advances in how apps and other digital tools can be used as an adjunct to clinician-led interventions. CBT practitioners may be interested in knowing that apps are available to support clients’ self-assessment of mental health problems, implementation of treatment techniques, taking fidelity ratings of therapy sessions, and monitoring of clinical outcomes over time (Pacheco and Scheeringa, 2022). Importantly, treatment adjuncts do not always have to occur concurrently with therapy. Digital tools such as online peer support platforms (Browne *et al*., 2022) and self-directed online booster sessions (Patterson *et al*., 2022) can be used after treatment ends. Browne *et al*. (2022) found that while not all participants reported regular use of the peer support platform, participants reported a benefit of simply knowing it is there. Such tools may therefore provide a sense of support which may be particularly valuable after treatment ends and clients are no longer receiving direct support from their therapist. Patterson *et al*. (2022) found that the offer of a post-treatment booster session was accepted by around a third of participants, who on average found the session helpful and felt it would help them manage symptoms of anxiety and depression in future. The use of digital technology in this way may open new possibilities to support clients in consolidating their therapy gains and maintaining their wellbeing after treatment ends, which previously would not have been practical.

The current challenge regarding digital adjuncts to treatment is a significant lack of studies that evaluate whether these ‘add-ons’ confer a benefit over usual care. Of the 59 apps reviewed in Pacheco and Scheeringa (2022), only 12 had supporting evidence from randomised controlled trials. The authors highlighted a need for more studies, and clearer information about empirical evaluations in app stores and descriptions. They also reported that few apps identified in their searches were in languages other than English. The development of innovations to be used alongside or following therapy is hugely valuable, but using robust study designs to evaluate their value is a vital next step before any wider implementation or dissemination can be considered. Where possible, these studies should include materials translated into other languages and people from groups under-represented in mental health services, including those who may be less familiar with or less confident in using digital tools.

### Remote treatment delivery

Articles in this special issue also show advances in our understanding of whether remote treatment via videoconferencing or telephone is effective. Examining data from two IAPT services, Nguyen *et al*. (2022) found that the shift to remote treatment delivery during the pandemic was not linked to significant worsening of clinical outcomes across any demographic group or presenting problem, although there were some indications that older clients, and clients with social anxiety or health anxiety, may have achieved less benefit from remote therapy. One IAPT service showed a significant increase in their overall recovery rate after moving to remote treatment provision. Porter *et al*. (2022) also found promising clinical outcomes in their evaluation of videoconferencing based therapy for young people in routine clinical practice, which showed medium to large effect sizes in anxiety and depression symptoms reduction and in progress towards personalised goals.

Given these positive findings, the studies also perhaps reflect advances in the skills and experience of practitioners who are delivering therapies using these formats. It is likely that many CBT practitioners had little or no experience of remote therapy delivery prior to the pandemic, so the present results are a credit to practitioners’ willingness to adapt in the face of significant adversity and change. Song and Foster (2022) document a positive shift in some therapists’ attitudes towards remote treatment. They do, however, also suggest that further therapist training in remote treatment delivery may still be needed. Although some guidance on adapting CBT procedures for remote sessions is available (e.g. Cromarty *et al*., 2020; Jassi *et al*., 2020; Murphy *et al*., 2020; Warnock-Parkes *et al*., 2020; Wild *et al*., 2020), there remain gaps, and further work to document and evaluate best practice would be beneficial, including disorder-specific or transdiagnostic CBT protocols, working with groups or family units, or specific client groups, such as adolescents or older adults. Behavioural experiments may pose the biggest challenge and require more substantial adaptation for remote delivery. Perhaps it is then reassuring to read the positive experiences of patients and therapists with regard to remote experiments (Song and Foster, 2022). However, this study also highlighted that some therapists found experiments harder to do. It would be interesting to explore how therapists deal with this, whether this results in fewer behavioural components in treatment, and perhaps even whether remote administration could facilitate ‘therapist drift’ (Waller and Turner, 2016) away from evidence-based therapy procedures.

The main evidence gap at present regarding our understanding of remote treatment is the lack of studies with appropriate control conditions. More robust study designs are needed, for example direct comparisons of in-person versus remote administration using the same therapeutic content. Clinicians need to better understand and be able to predict who does or does not benefit from inperson versus remote delivery. Telephone-delivered treatment was included in the Nguyen *et al*. (2022) study, and although it was not examined directly, the results from Service B, which replaced in-person work primarily with telephone sessions during the pandemic, indicated that clinical outcomes improved for low-intensity CBT interventions, but declined for high-intensity interventions. This warrants further investigation as it may aid the interpretation of reviews of telephone treatments (e.g. Coughtrey and Pistrang, 2018). Comparisons of telephone versus videoconference delivery would also be valuable.

### User experiences

The present articles also demonstrate advances in our knowledge of, and approaches to, user experiences of digital CBT. Many of the studies (Browne *et al*., 2022; Li *et al*., 2022; Patterson *et al*., 2022; Porter *et al*., 2022; Song and Foster, 2022) have incorporated at least an element of user feedback, which is perhaps a positive reflection of the value placed on this information as well as its importance in the development, refinement or delivery of interventions. Taking this one stage further, Li *et al*. (2022) demonstrate in their study advances in how co-design principles are now being used to involve the ‘end-users’ of a digital product in its development from the outset. This study describes the iterative process used, involving adolescents, parents and mental health professionals in the design of a CBT app, and highlights differences in preferences among end-users that need to be accounted for in app development (e.g. adolescents prefer an app they can use autonomously, while mental health professionals desire an app they can use as an adjunct to therapy). Li *et al*. (2022) highlight that co-design does not appear to be common practice, or at least that the reporting of co-design or user involvement procedures is lacking, citing a recent review that found this occurred in less than 30% of preventative mental health interventions for young people (Bergin *et al*., 2020). There is clearly scope for further improvement in the extent of meaningful user involvement as well as how this is described in the literature.

Another significant challenge is to ensure that the data gathered from evaluations of user experiences is representative. We must continue to improve our efforts to avoid sampling biases such that only the experiences of users who completed an intervention, or who were sufficiently motivated to attend user groups or complete surveys, are heard. Browne *et al*. (2022) and Patterson *et al*. (2022) made specific efforts to obtain feedback from those who had dropped out or were no longer using the intervention. This was informative in revealing the range of reasons that might be behind drop-out (such as forgetting about it, lack of time, not finding it helpful, or no longer needing it due to feeling better), but more could be done in future studies to examine specifically the experiences of those who disengage or stop using a particular intervention. This is critical to ensure that studies of user experiences or client satisfaction are not biased towards positive responses or missing the views of underrepresented groups.

### CBT research

The advances we have considered so far focus primarily on how digital methods can be used to deliver or enhance CBT interventions; but this set of articles perhaps also reflects advances in the use of digital tools to support service evaluation and research in CBT. Online administration of outcome questionnaires, surveys and focus groups are all demonstrated within this set of articles, which illustrate progress in how technology is being used to facilitate studies, and the collection of routine outcome data. It is promising to see that many of the present articles report on work conducted in routine clinical practice (Browne *et al*., 2022; Nguyen *et al*., 2022; Porter *et al*., 2022; Song and Foster, 2022), and it may well be the case that these studies have been made more achievable due to the technological advances that support data collection. Beyond advancing digital CBT, the studies serve as valuable examples of how to collect data in routine care and use this data collection to iteratively improve services.

However, as we make greater use of digital technology to develop, deliver and research CBT interventions, it is critical to ensure that these methods do not exclude people. With regard to psychological therapies, ‘digital exclusion’ occurs when people are not equally able to access mental health services, receive psychological support, or participate in research, because they do not have access to the internet or relevant devices, or they do not have the skills or confidence to use this technology. There is evidence that the latter may be worsened by mental health problems (Greer *et al*.,2019). This issue also applies to CBT practitioners, who may feel hesitant or lacking in skills to use digital technology effectively in their work. Developing a fuller understanding of digital exclusion in relation to CBT practice and research, and finding ways to minimise this inequality, is an advance much needed in the field. Lastly, another topic that warrants further research is the potential negative effects of delivering CBT methods digitally, which should be explored and reported more extensively to ensure patient safety and minimise unwanted and adverse events (Rozental *et al*., 2014).

## Where next?

Drawing on the advances and possible next steps identified among these articles, we now consider the broader digital CBT picture. Below we present our thoughts and suggestions for six key directions for future research that we believe would be most beneficial for continuing to advance the field.

### Using robust study designs to evaluate and optimise digital tools

High-quality empirical evaluations are currently lacking in various areas of digital CBT. This would include, but is not limited to, randomised controlled trials, and a range of study designs are possible. In particular, the use of appropriate control groups, including comparison with in-person treatment, and/or experimental manipulations would be valuable. Study designs that incorporate a focus on participants who disengage, drop out, or do not benefit from digital CBT tools are also much needed to help optimise digital interventions to enhance user engagement and outcomes.

### Translating and culturally adapting digital tools and practices

One of the potential benefits of digital interventions and tools is their global reach and ability to improve access to CBT among those who may have difficulty accessing traditional mental health services. Further advances in translating and culturally adapting digital CBT tools are needed, as well as evaluations of their effectiveness. This would facilitate both the international dissemination of CBT, and access to services in linguistically and culturally diverse areas.

### Understanding and addressing digital exclusion

The field would benefit from a greater understanding of digital exclusion in relation to CBT. This would provide a foundation on which to explore ways to meaningfully address this issue and ensure that digital CBT provision is accessible and equitable.

### Exploring, reporting and addressing possible negative effects

CBT and psychological treatments in general have clear benefits for those suffering from mental health issues. However, in some cases, patients experience unwanted and adverse events, such as clinical deterioration or feelings of hopelessness. Although the topic has gained more attention over the last decade, researchers should investigate, report and address the occurrence and characteristics of potential negative effects more systematically. This is of particular relevance for digital CBT, where delivery is often new and unexplored.

### Improving user involvement in design and evaluation

Collaboration is a central principle of CBT, and this ethos needs to be applied more consistently when digital tools are being designed, enhanced and evaluated. Integrating meaningful involvement of clients, clinicians, service managers and other potential end-users is critical to ensure that digital tools are user-friendly, practical and clinically beneficial. Such involvement is likely to involve eliciting user preferences, but may go further, having end-users play a more active role in the design or implementation of evaluation studies. This is a crucial step towards more effective use of technology to provide or enhance treatment, whether as adjuncts to therapist-delivered CBT, support following treatment, or standalone tools.

### Addressing the implementation gap for digital tools

Much time, effort and money is spent on developing new digital tools to deliver or enhance treatment, but many do not progress beyond this stage, falling into the ‘implementation gap’. This means that many helpful innovations may exist but are not able to benefit our clients. Greater focus on and funding for work to implement and embed digital approaches within services, disseminate them more widely, and support their ongoing upkeep and sustainability is needed.

## Conclusion

The articles in the special issue provide a snapshot of how advances are being made across areas of digital CBT including assessment, treatment delivery and evaluation. They reflect the rapidly developing knowledge base in this field and point to many key directions for future research. This article has aimed to synthesise and reflect on these advances, considering where the field is currently, and where some of the next advances might be achieved. There are many exciting opportunities to take the present work forward and progress the field more broadly, including those that can be achieved within routine clinical practice. CBT has a long history of evolving and adapting to meet new clinical challenges. Harnessing the power of digital technology is a promising route to continue this progression, one in which all practitioners, researchers and users of CBT could play a role.

## Data Availability

Data availability is not applicable to this article as no new data were created or analysed in this study.

## Key learning aims

(1) To gain an overview of the articles in the special issue and an understanding of the advances in digital CBT they represent.
(2) To understand how the advances suggested by the present studies could be taken forward and extended.
(3) To consider key future directions for further advances in digital CBT.

## Key practice points

(1) Practitioners should closely consider the underpinning evidence when considering which mental health apps to recommend to clients or use within treatment.
(2) Practitioners may wish to reflect on their skills and development needs regarding remote treatment delivery and seek further supervision or training as appropriate.
(3) Given the need for further research and service evaluation in digital CBT, practitioners may wish to seek opportunities to contribute to such projects as part of their continuing professional development.
